# Supplementary material for: Linear leaky-integrate-and-fire neuron model based spiking neural networks and its mapping relationship to deep neural networks
Source: Front Neurosci. 2022 Aug 24;16:857513. doi: 10.3389/fnins.2022.857513 (PMC9448910; doi:10.3389/fnins.2022.857513)
Supplement: Supplementary file 1 [file Presentation_1.zip › frontiers_SupplementaryMaterial.pdf]

frontiers

Supplementary Material

1 THE LINEAR LIF MODEL

1.1 The basic equation of Linear LIF model

The essence of the LIF model is the parallel connection between membrane resistance R_M and membrane capacitance C_M. Assuming membrane resistance R_M is voltage-independent, $R_M = C$ is a constant. According to Ohms law, the current through the membrane resistance is V/R_M. Based on the definition of capacitor $C = Q/U$ (where Q is the amount of charge, and U is voltage), the membrane capacitive current is $I_{C_m} = C_M dV/dt$. According to the law of current conservation, we can get the basic equation of the LIF model:

$$C_m \frac{dV(t)}{dt} + \frac{V(t) - V_0}{R_m} = I_{inj}, \tag{S1}$$

where C_M is membrane capacitance, R_M is the membrane resistance and I_{inj} is the input current.

The integral factor method is a conventional method for solving linear first-order differential equations. Based on this method, Eq. S1 can be further reduced to the equation of membrane potential varying with the input current, as shown in Eq. S2.

$$V(t) = e^{-\frac{t-t_0}{\tau_m}} \left[\int_{t_0}^{t} \frac{I_{inj}(t')}{C_m} e^{\frac{t'-t_0}{\tau_m}} dt' + V(t_0) \right] \tag{S2}$$

where $\tau_m = C_m \cdot R_m$ is the membrane time constant, $I_{inj}(t)$ is the input current, C_m is th membrane capacitor, R_m is the membrane resistor and $V(t_0)$ is the initial membrane potential.

1.2 Membrane potential under spiking threshold

In spiking neural networks, the most common coding method is frequency coding. That is, the pixel value is encoded into a periodic spike sequence. If there is a pulse, it is 1. Otherwise, it is 0. The encoding time (time window) is T_w, sampling frequency is the maximum frequency of encoding $R_{max} = 1/\Delta t$. Assuming that the pixel value to be encoded is $X_i\ in[0, 1]$, the encoded spike sequence can be expressed as:

$$I_{inj} = \omega_i \sum_{j=1}^{N} \delta \left(t - j\frac{1}{x_i} \right) \tag{S3}$$

where, $\delta(\ )$ is the delta function, $N = x_i \cdot T_w = \sum I_{inj}$ is the number of spikes in time windows T_w.

Eq. S4 expresses the membrane potential with a periodic spike signal.

$$V(t = n/x_i) = \frac{\omega_i}{C_m} \cdot \left(1 + e^{-\frac{T_i}{\tau_m}} + e^{-\frac{2T_i}{\tau_m}} + \cdots + e^{-\frac{(n-1)T_i}{\tau_m}} \right) \tag{S4}$$

when the neuron receives a spike from presynaptic, the membrane potential will accumulate. Without spike input, the membrane potential will decay exponentially. According to the summation formula of equal ratio sequence, we further simplify Eq. S4 and obtain the variation equation of membrane potential.

$$V\left(t=n/x_i\right)=\frac{\omega_i}{C_m}\cdot\frac{1-e^{-\frac{(n-1)T_i}{\tau_m}}\cdot e^{-\frac{T_i}{\tau_m}}}{1-e^{-\frac{T_i}{\tau_m}}}=\frac{\omega_i}{C_m}\cdot\frac{1-e^{-\frac{nT_i}{\tau_m}}}{1-e^{-\frac{T_i}{\tau_m}}} \tag{S5}$$

1.3 Membrane potential change with spiking threshold

The membrane potential accumulates with inputs $I(t)$. Once the membrane potential $V(t)$ exceeds the spiking threshold V_{th}, the neuron fires an action potential, and the membrane potential $V(t)$ goes back to the resting potential V_0. The LIF model is a typical nonlinear system. Three discrete equations can describe the charge, discharge, and fire of the LIF model:

$$\begin{aligned} H(t) &= f(V(t-1), I(t)) \\ S(t) &= \Theta(H(t)-V_{th}) \end{aligned} \tag{S6}$$

where the $H(t)$ is the membrane potential before spike, $S(t)$ is the spike train and $f(V(t-1), I(t))$ is the update equation of membrane potential.

Once the membrane potential reaches the spiking threshold, an action potential will be exceeded. Then the membrane potential will be reset: 'reset to zero,' used, e.g., in **?**, reset the membrane potential to zero. 'linear reset' retains the attenuation term that exceeds the threshold:

$$V(t)=\begin{cases} H(t)\cdot(1-S(t)) & \text{reset to zero} \\ H(t)\cdot(1-S(t))+(H(t)-V_{reset})\cdot S(t) & \text{linear mode} \end{cases} \tag{S7}$$

The LIF neuron model with 'linear reset mode' is named the linear LIF model. **?** and **?** analyzed the difference between these two MP reset modes and chose the linear LIF model for simulation. We analyze the two models from the perspective of physics and information theory and determine the advantages of the linear LIF model. For the first reset mode, the membrane potential of the LIF model does not satisfy the law of conservation of energy. There are two parts of membrane potential attenuations: 'leaky', the attenuations as the form of conductance in the circuit which keeps the nonlinear dynamic properties. The other part is that when the action potential is exceeded, the membrane potential exceeding the spike threshold will be lost directly, resulting in energy non-conservation. From the perspective of information, the linear LIF neuron model maintains the nonlinearity of the model and retains the completion of information to the greatest extent.

2 THE MAPPING RELATIONSHIP BETWEEN LINEAR LIF MODEL AND RELU-AN MODEL

In this chapter, we derive the parameter mapping relationship between the linear LIF model and the artificial neuron model (ReLU-AN) model. The relationship between input and output in the artificial neuron model can be expressed as:

$$y_j=f(\sum_i \omega_{ji}x_i+b_j) \tag{S8}$$

where ω_{ji} is the connection weight between presynaptic neuron j and postsynaptic neuron i. b_i is the bias of unit i, and $f(\ )$ is the activation function.

Here we give the parameter mapping we established in Tab.S1, which will be discussed in detail later in this paper.

Parameter of ReLU		Params of linear LIF	
**Symbol**	**Description**	**Symbol**	**Description**
ω	Connection weight	ω	Synaptic weight
b	Bias	$\frac{-\sum\omega}{R_m C_m \cdot \ln(1-\sum\omega/(V_{th}C_m))}$	
k	Slope of activation function	$1/V_{th}C_m$	

Table S1. Parameter mapping between ReLU-AN model and linear LIF model

2.1 Mapping of the weights

We assume that the spiking frequency of the input signal is f_j and the amplitude is 1, and then the signal can be expressed as:

$$I_{inj}{}_k^l(t) = \sum_{i=1}^{n^l} \omega_i \cdot \sum_{j=1}^{N_i^l} \delta(t - j\frac{1}{f_i}) \tag{S9}$$

The ω_i is the synaptic weight between presynaptic neuron i and post-synaptic neuron, n^l represent the number of neuron in layer l, j represents the j_{th} action potential in the input spike train, N_i^l is the number of action potentials, and T_w is the time windows of simulation.

According to frequency coding, the number of action potentials can be solved by the product of spike frequency and coding time: $N_i^l = f_i \cdot T_w = \sum I_{inj}{}_k^l$. Compared with the weight integration process in ANNs, we integrate the input signal $I_{inj}{}_k^l(t)$ in the time window $[0, T_w]$ and obtain:

$$\int_0^{T_w} I_{inj}{}_k^l dt = \sum_{i=1}^{n^l} \omega_i \cdot \sum_{j=1}^{N_i^l} \int_0^{T_w} \delta(t - j\frac{1}{f_i})dt = T_w \cdot \sum_{i=1}^{n^l} \omega_i f_i \tag{S10}$$

2.2 Mapping of the bias

Due to the attenuation of membrane potential in the Linear LIF model, it is possible that even if the encoding time is long enough, neurons may not generate an action potential. If there is an action potential output within the encoding time, it must satisfy:

$$V\left(N/f_i\right) \geq V_{th} \tag{S11}$$

Assuming that there are N action potentials in the coding time T_w, we can get:

$$\frac{\sum_{i=1}^{n^l} \omega_i}{C_m} \cdot \frac{1 - e^{-\frac{NT_i}{\tau_m}}}{1 - e^{-\frac{T_i}{\tau_m}}} \geq V_{th} \tag{S12}$$

Using $T_W f_i \geq N$ and leads to:

$$e^{-\frac{T_W}{\tau_m}} \leq e^{-\frac{NT_i}{\tau_m}} \leq 1 - \frac{C_m V_{th}\left(1 - e^{-\frac{T_i}{\tau_m}}\right)}{\sum_{i=1}^{n^l} \omega_i} \tag{S13}$$

Simplify the formula and we can get:

$$e^{-\frac{T_i}{\tau_m}} \geq 1 - \frac{\sum_{i=1}^{n^l} \omega_i \cdot \left(1 - e^{-\frac{T_W}{\tau_m}}\right)}{C_m V_{th}} \tag{S14}$$

By taking logarithms on both sides of the equation, we can get:

$$-\frac{T_i}{\tau_m} \geq ln\left[1 - \frac{\sum_{i=1}^{n^l} \omega_i \cdot \left(1 - e^{-\frac{T_W}{\tau_m}}\right)}{C_m V_{th}}\right] \tag{S15}$$

Transferring the period into frequency, the minimum input is given by

$$\sum_{i=1}^{n^l} \omega_i f_i \geq -\frac{\sum_{i=1}^{n^l} \omega_i}{\tau_m ln\left[1 - \frac{\sum_{i=1}^{n^l} \omega_i \cdot \left(1 - e^{-\frac{T_W}{\tau_m}}\right)}{C_m V_{th}}\right]} \tag{S16}$$

Assume the encoding time meets the ideal condition, $T_W \to \infty$. Then $e^{-T_W/\tau_m} \to 0$, the formula can be further simplified to:

$$\sum_{i=1}^{n^l} \omega_i f_i \geq -\frac{\sum_{i=1}^{n^l} \omega_i}{\tau_m ln\left(1 - \frac{\sum_{i=1}^{n^l} \omega_i}{C_m V_{th}}\right)} \tag{S17}$$

2.3 Mapping of activation function

The activation function determines the relationship between the integrated input and output. We focus on the non-negative and linear relationship of ReLU. For nonnegative, the output of the Linear LIF model is based on the number of action potentials, which is a non-negative value.

We assume that the membrane potential reaches the spiking threshold after the n action potential, and the neuron generates action potentials, that is:

$$V\left(t=n/x_i\right)=\frac{\sum_{i=1}^{n^l}\omega_i}{C_m}\cdot\frac{1-e^{-\frac{nT_i}{\tau_m}}}{1-e^{-\frac{T_i}{\tau_m}}}\geq V_{th} \tag{S18}$$

The above formula can be reduced to:

$$1-\frac{V_{th}C_m\left(1-e^{-\frac{T_i}{\tau_m}}\right)}{\sum_{i=1}^{n^l}\omega_i}\geq e^{-\frac{nT_i}{\tau_m}} \tag{S19}$$

By taking logarithms on both sides, we can get:

$$\frac{n}{\tau_m f_i}\geq -ln\left[1-\frac{V_{th}C_m\left(1-e^{-\frac{T_i}{\tau_m}}\right)}{\sum_{i=1}^{n^l}\omega_i}\right] \tag{S20}$$

With $f_o = f_{in}/\lfloor n \rfloor$, the spike frequency of output spike train can be expressed by

$$\frac{f_i}{n}\leq\frac{1}{-\tau_m ln\left[1-\frac{V_{th}C_m\left(1-e^{-\frac{T_i}{\tau_m}}\right)}{\sum_{i=1}^{n^l}\omega_i}\right]} \tag{S21}$$

Using $ln(1-x)\approx x$, $e^{-x}\approx x$ and $1-e^{-\frac{T_i}{\tau_m}}\rightarrow 0$, we can get

$$f_o=\frac{f_i}{n}\leq\frac{1}{V_{th}C_m}\sum_{i=1}^{n^l}\omega_i f_i \tag{S22}$$
